# Effect of α-Glucosylation on the Stability, Antioxidant Properties, Toxicity, and Neuroprotective Activity of (–)-Epigallocatechin Gallate

**DOI:** 10.3389/fnut.2019.00030

**Published:** 2019-03-22

**Authors:** Jose L. Gonzalez-Alfonso, Pablo Peñalver, Antonio O. Ballesteros, Juan C. Morales, Francisco J. Plou

**Affiliations:** ^1^Instituto de Catálisis y Petroleoquímica, CSIC, Madrid, Spain; ^2^Instituto de Parasitología y Biomedicina López–Neyra, CSIC, Granada, Spain

**Keywords:** glycosylation, tea polyphenols, antioxidants, catechins, neuroprotective properties

## Abstract

(–)-Epigallocatechin gallate (EGCG), the predominant catechin (≥50%) in green tea (*Camellia sinensis*), displays several bioactive properties but its stability and bioavailability are low. In this work, the properties of two α-glucosyl derivatives of EGCG (3′- and 7-O-α-D-glucopyranoside), obtained by enzymatic synthesis, were assessed. The α-glucosylation enhanced the pH and thermal stability of EGCG. The analysis of scavenging activity toward ABTS^·^+ radicals showed that the α-glucosylation at C-7 of A-ring caused a higher loss of antioxidant activity compared with the sugar conjugation at C-3′ of B-ring. The 3′-glucoside also showed higher potential to alleviate intracellular reactive oxygen species (ROS) levels and to boost REDOX activity. The toxicity of EGCG and its monoglucosides was tested in human SH-S5Y5 neurons, RAW 264.7 macrophages, MRC5 fibroblasts, and HT-29 colon cancer cells. Interestingly, the 3′-O-α-D-glucoside increased the viability of neural cells *in vitro* (2.75-fold at 100 μM) in the presence of H_2_O_2_, whilst EGCG gave rise only to a 1.7-fold enhancement. In conclusion, the α-glucoside of EGCG at C-3′ has a great potential for nutraceutical, cosmetic and biomedical applications.

## Introduction

Plant polyphenols are gaining relevance due to their capacity to delay the appearance of certain degenerative diseases and pathological processes such as Alzheimer's and Parkinson's diseases, schizophrenia, cancer, chronic inflammatory disease, atherosclerosis or myocardial infarction (1–3). Their action is based on the enhancement of the antioxidant system due to their ability to reduce the level of reactive oxygen species (ROS) (4). Many polyphenols are lipophilic scaffolds with rapidly conjugated phenolic OHs that exhibit poor absorption *in vivo*, giving rise to a very low concentration in the circulatory streams (5).

Several polyphenols appear glycosylated in nature (4, 6) and the sugar moiety seems to play a major role in their solubility (7), partition coefficient (8), protection from oxygen, pH, temperature and/or light (9), absorption (10, 11), bioavailability (12), and bioactivity (13). Several studies demonstrated that glycosylation facilitates the diffusion of polyphenols into intestinal enterocytes (12, 14). Other investigations have shown that deconjugation of the glycosyl moiety of glycosylated flavonoids favors cellular uptake by enterocytes (15, 16). Despite this controversy in the role of glycosylation on bioavailability, there is some consensus that glycosylation increases the stability of polyphenols during gastrointestinal transit after ingestion (17) and also during storage (18). In fact, glycosylation is being exploited as a tool to improve the properties of polyphenols (7, 19–22). Enzymatic synthesis is gaining importance due to its selectivity and the environmentally friendly reaction conditions (23–25).

(–)-Epigallocatechin gallate (EGCG) is the predominant catechin (≥50%) in green tea (*Camellia sinensis*). It possesses antioxidant (26), antihypertensive (27), antitumoral (28, 29), bactericidal (30), and anti-inflammatory (31) bioactivity, among others. However, EGCG undergoes rapid degradation in aqueous solutions (32) resulting in a low bioavailability (33). The two main processes involved in the instability of EGCG are epimerization and oxidative coupling (34). In order to increase its stability and bioavailability (35), and to reduce its astringency for food applications (36), the glycosylation of EGCG has been explored by several groups, mostly by the use of enzymatic catalysis (37–39). Recently, our group reported the enzymatic synthesis of various α-glucosyl derivatives of EGCG by a transglycosylation reaction catalyzed by a cyclodextrin glucanotransferase (CGTase, EC 2.4.1.19) (40). Two main α-D-glucosides of EGCG were isolated and chemically characterized: EGCG 3′-O-α-D-glucopyranoside (***1***) and EGCG 7-O-α-D-glucopyranoside (***2***).

In the present work, we have analyzed the effect of α-glucosylation on several properties of EGCG, in particular the pH and thermal stability, the antioxidant and REDOX activities, the toxicity toward several cell lines and the neuroprotective activity. Consequently, the influence of the position of glycosylation on such properties was assessed.

## Materials and Methods

### Enzyme and Reagents

(-)-Epigallocatequin gallate (EGCG) was acquired from Zhejiang Yixin Pharmaceutical Co. (Zhejiang, China). Toruzyme 3.0L, a commercial preparation of cyclodextrin glucanotransferase (CGTase) from *Thermoanaerobacter* sp., was kindly provided by Novozymes. Partially hydrolyzed starch from potato (Passelli SA2) was from Avebe (Foxhol, The Netherlands). ABTS [2,2′-azino-bis(3-ethylbenzothiazoline-6-sulphonic acid)] and (R)-Trolox (6-hydroxy-2,5,7,8-tetramethylchroman-2-carboxylic acid) were purchased from Sigma Aldrich. All other reagents and solvents were of the highest available purity and used as purchased.

### Stability Assays

EGCG and its glucosylated derivatives were dissolved at 4 mg/mL in 20 mM sodium phosphate buffer (pH 6.7) and incubated at 60°C. At intervals, aliquots of 150 μL were withdrawn, diluted 2-fold with water and passed through nylon filters (13 mm, 0.45 μm). The remaining concentrations of EGCG or its glucoside were analyzed by HPLC.

### Trolox Equivalent Antioxidant Capacity (TEAC) Assay

The ABTS^·+^ was generated from ABTS solution (7 mM) with potassium persulfate (2.45 mM) for 15 h. The radical cation absorbed at 734 nm and was stable for 2 days. ABTS^·+^ was diluted in ethanol to 0.7 ± 0.02 absorbance units at 734 nm. Addition of antioxidants to the pre-formed radical cation reduces it to ABTS thus decreasing the absorbance. Twenty microliter of antioxidant solution (between 20 and 210 μ M) was added to 230 μL of adjusted ABTS^·+^ solution. The decrease of absorbance of the ABTS^·+^ solution was monitored at 734 nm during 6 min using a microplate reader (model Versamax, Molecular Devices). The decrease of absorbance was determined measuring the area under the curve. (R)-Trolox was used as a reference antioxidant. The TEAC value was expressed as the concentration (μM) at which the compound decreases the same absorbance as 1 μM (R)-Trolox.

### Cell Cultures

SH-S5Y5 neurons were cultured in collagen-pretreated petri-dishes with DMEM-F12 medium supplemented with penicillin/streptomycin and 10% inactivated fetal bovine serum (iFBS). RAW 264.7 macrophages and HT-29 colon cancer cells were cultured in DMEM high glucose medium supplemented with penicillin/streptomycin and 10% iFBS. MRC5 were cultured in DMEM low glucose medium supplemented with glutamine, penicillin/streptomycin and 10% iFBS.

### Cell Viability Assays

Neuron assays were done in collagen-pretreated 96 well plates by seeding 2 × 10^4^ neurons per well in a 100 μL volume and with 24 h of incubation before the compound addition. Macrophage assays were done in 96 well plates by seeding 2.5 × 10^4^ macrophages per well in a 100 μL volume with 4 h of incubation before the compound addition. MRC5 and HT-29 assays were done in 96 well plates by seeding 5 × 10^4^ cells per well in a 100 μL volume and with 24 h of incubation before the compound addition. Tested compounds dissolved in DMSO were then added at different final concentrations (100, 10, and 1 μM) to determine compound toxicity. Final DMSO percentage in each cell was adjusted to 1%. Cell viability was evaluated 24 h (SH-SY5Y and RAW 264.7 cells) or 48 h (MRC5 and HT-29 cells) after compounds addition by mitochondrial MTT assay, according to manufacturer.

### Measurement of Reactive Oxygen Species (ROS)

Reactive oxygen species (ROS) levels were evaluated using the ROS-sensitive H_2_DCFDA staining method (Sigma, St. Louis, MO, USA). The intracellular ROS level was determined on SH-SY5Y neuroblastoma cells that were cultured, plated and compound-treated as described previously for the cell viability assay. The protective effect of the EGCG derivatives on H_2_O_2_-induced oxidative stress was assayed after a short pre-incubation time of the compounds (2 h) followed by a short incubation with H_2_O_2_ (100 μM, 2 h). The intracellular ROS generation of each compound alone, without H_2_O_2_ treatment, after 6 h of incubation, was also evaluated. Following treatments, the medium was removed and incubated with 25 μM H_2_DCFDA for 2 h at 37°C in the dark. H_2_DCFDA, a cell permeable non-fluorescent, is de-esterified intracellularly and turns to the highly fluorescent permeant molecule 2,7-dichlorofluorescein (DCF) in the presence of intracellular ROS upon oxidation. Fluorescence intensity was measured at an excitation wavelength of 485 nm and an emission wavelength of 530 nm using a multimode microplate reader (TECAN, Männedorf, Switzerland).

### Mitochondrial Oxidation–Reduction (REDOX) Activity

The analysis of REDOX activity was performed using the fluorogenic oxidation-reduction indicator Resazurin (Life Techonologies Inc., Rockville, MD, USA). The REDOX activity level was determined on SH-SY5Y cells that were cultured, plated and compound-treated as described previously. After treatments, resazurin dissolved in water at a final concentration of 5 μM was added to the wells, and the fluorescence intensity was examined at an excitation of 530 nm and an emission of 590 nm. The plate was incubated for 2 h, and then fluorescence was measured using a multimode microplate reader (TECAN, Männedorf, Switzerland).

### Neuroprotective Properties

EGCG and the corresponding glucosides were assayed *in vitro* in cell cultures to determine their neuroprotective activity. SH-S5Y5 neurons were determined on SH-SY5Y cells that were cultured, plated and compound-treated as described previously. EGCG and its glucosides dissolved in DMSO were added at three concentrations (1, 10, and 100 μM) and incubated for 10 min before the addition of hydrogen peroxide (100 μM). Cell viability was evaluated 24 h after compound addition by mitochondrial MTT assay. Neuron recovery was calculated by normalizing the results from H_2_O_2_-neuron viability to the H_2_O_2_ positive control.

### Statistical Analysis

For the determination of antioxidant capacity (TEAC assay), experiments were performed in triplicate. The standard deviations of TEAC values were calculated from the slope of linear regressions of the curves representing decrease of absorbance vs. concentration. The significant differences between the values were calculated with a *t*-test of slopes and their standard deviations, considering *n* the number of linear regression points.

For the cell viability assays, analysis of ROS, mitochondrial oxidation-reduction activity and neuroprotective activity, averages and standard deviations of at least eight different readings from various experiments were calculated. Welch's *t*-test for samples with unequal variance (previously tested by one way ANOVA in SigmaPlot 13.0) was made to perform the statistical analysis, considering significant differences when *p* < 0.05.

## Results and Discussion

### EGCG Glucosylation and Effect on Antioxidant Properties

**Figure 1:**
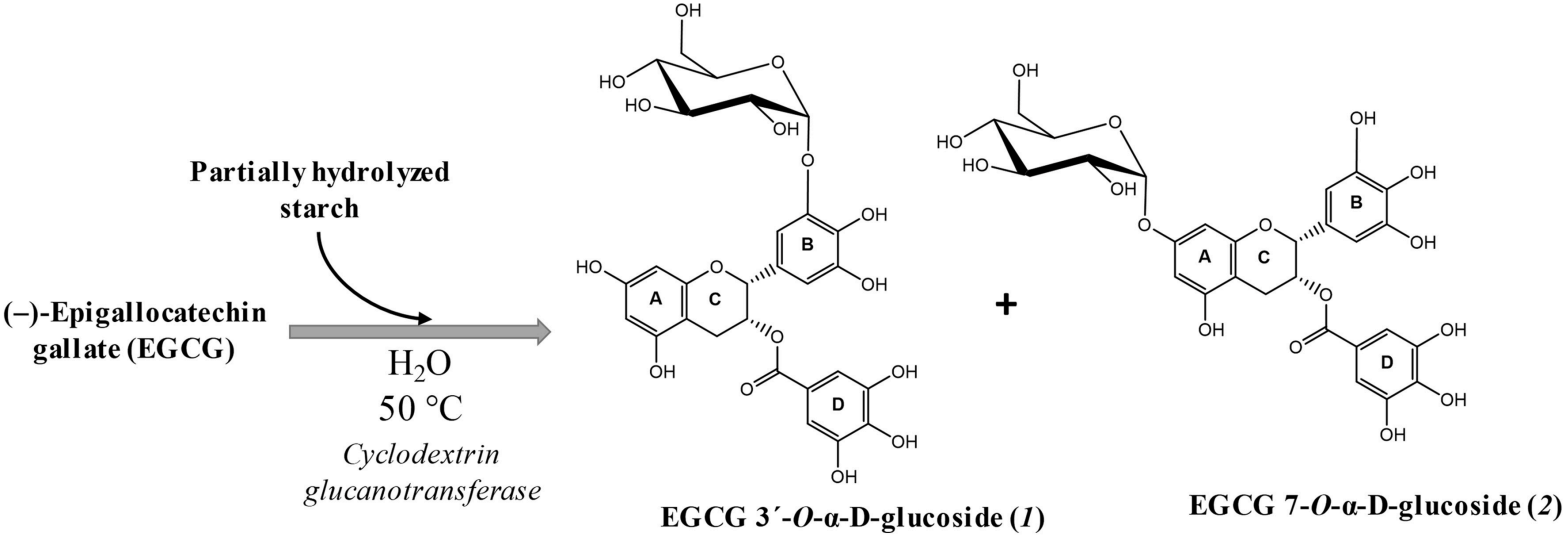
Scheme of the glucosylation of EGCG and the structure of the two main products: epigallocatechin gallate 3′-*O*-α-D-glucopyranoside **(*1*)** and epigallocatechin gallate 7-*O*-α-D-glucopyranoside **(*2*)**.

The synthesis of various α-glucosyl derivatives of (–)-epigallocatechin gallate (EGCG) was performed following a previous work developed in our laboratory (40). The reaction takes place at 50°C catalyzed by cyclodextrin glucanotransferase (CGTase) from *Thermoanaerobacter* sp., using hydrolyzed potato starch as glucosyl donor (Figure 1). The reaction was performed in water (no buffer), as the maximum stability of EGCG was found in this solvent (40). Two main monoglucosides were the main products and were chemically characterized by combining MS 2D-NMR methods. The major derivative was epigallocatechin gallate 3′-O-α-D-glucopyranoside (***1***) and the minor epigallocatechin gallate 7-O-α-D-glucopyranoside (***2***).

**Figure 2:**
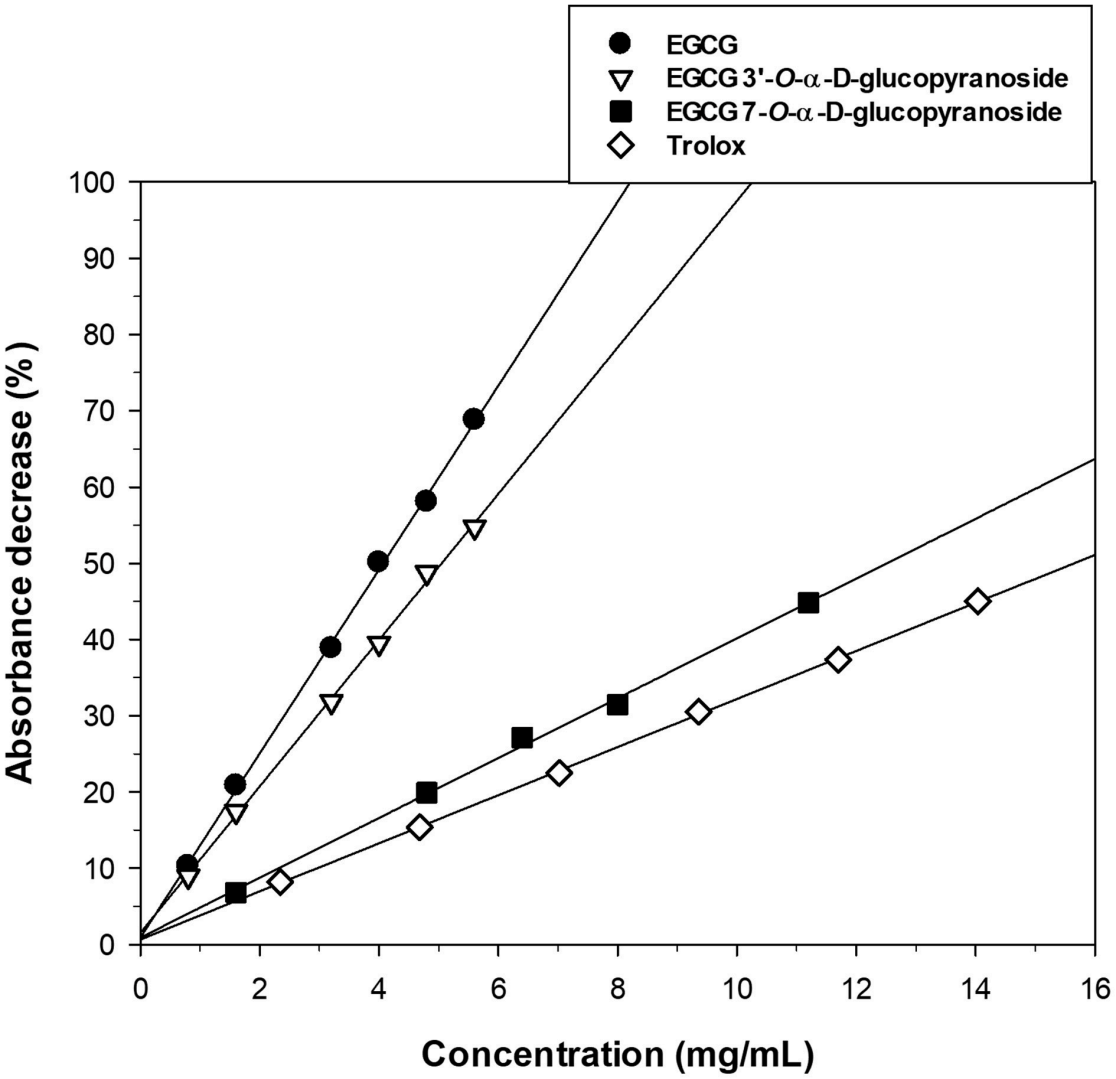
Effect of EGCG and its α-glucosides ***1*** and ***2*** on ABTS^·+^ reduction. In the assay, Trolox was used as antioxidant reference compound.

We studied the antioxidant activity of the two glucosylated derivatives by the TEAC assay to assess the role of the different phenolic groups on the EGCG properties. The results of the assay are represented in Figure 2. The incorporation of a α-glucosyl moiety to the position 7 of A-ring caused a higher loss of antioxidant activity than in position 3′ of B-ring. The TEAC values, calculated from the slopes of linear regressions of Figure 2, are summarized in Table 1. In all cases the TEAC values were lower than that obtained for Trolox.

**Table 1:** TEAC values of EGCG and its α-glucosides.

**Compound**	**Slope of linear regression**	**R^**2**^**	**TEAC**
Trolox	3.22 ± 0.02	0.996	1.00 ± 0.02
EGCG	12.1 ± 0.1	0.999	0.27 ± 0.02
EGCG 3′-*O*-α-D-glucopyranoside	9.97 ± 0.20	0.996	0.32 ± 0.02^*^
EGCG 7-*O*-α-D-glucopyranoside	4.04 ± 0.20	0.999	0.80 ± 0.04^*^

*The data is expressed as mean ± SD (n = 6*,

* *p < 0.01 vs. EGCG)*.

As shown in Figure 2, the glucosylation at the 3′-position has a slight influence on the scavenging activity of EGCG toward ABTS^.+^ radicals. In this context, it has been reported that the *ortho*-trihydroxyl group (at positions C-3′,−4′, and−5′) at B-ring and the gallate moiety at C-3 of A-ring are the most important structural features for scavenging free radicals by EGCG (38, 41). Our results compare well with those described by Nanjo et al. using the DPPH radicals assay (41, 42) However, it must be considered that the free radical scavenging capacity of tea catechins and their derivatives is radical-dependent (32). In the case of DPPH radical scavenging, it has been demonstrated that both the 4′-OH at B-ring and the 4″-OH at the galloyl moiety are essential to maintain antioxidant activity (39, 43).

### Stability of EGCG Glucosides

It is well-reported that the stability of EGCG in aqueous solutions is rather limited (37, 44, 45). The two main processes involved in the degradation of EGCG are epimerization and oxidative coupling (34). The stability of EGCG is concentration-dependent and can be also influenced by temperature, pH and the amount of oxygen in the solution, among other parameters (32).

**Figure 3:**
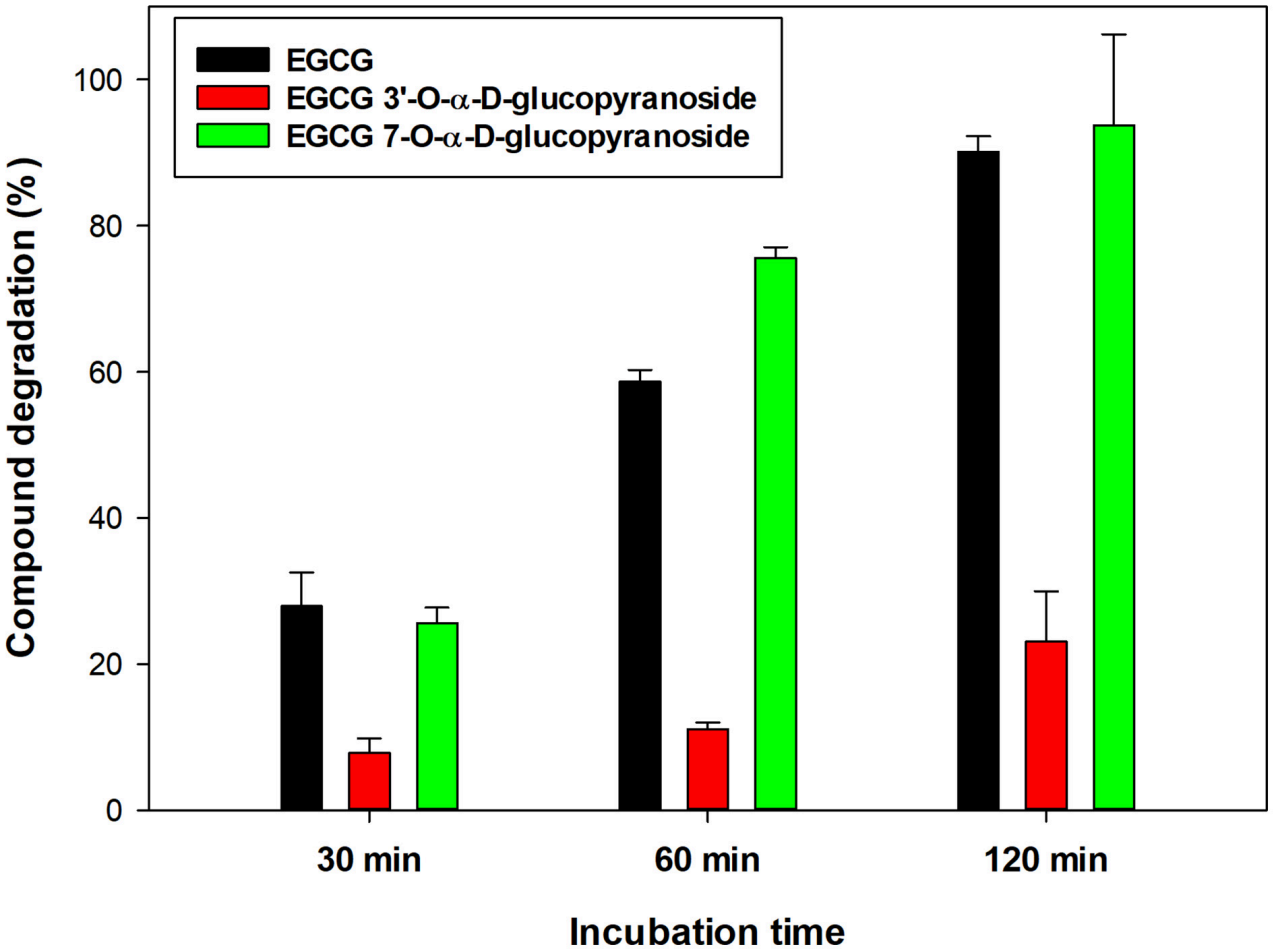
Relative degradation of EGCG and its α-glucosides ***1*** and ***2*** under standard conditions: [Compound] = 4 mg/ml, 20 mM sodium phosphate buffer (pH 6.7), 60°C.

The stability of EGCG and its two monoglucosides in a buffered solution was comparatively studied. The compounds (4 mg/mL) were dissolved in 20 mM phosphate buffer (pH 6.7) and incubated at 60°C. As shown in Figure 3, the EGCG was degraded about 4-fold faster than the monoglucoside ***1***. The degradation process was concomitant with the appearance of (**–**)-gallocatechin gallate (GCG) as a result of EGCG epimerization (data not shown). The color of the solutions became brown upon incubation, as a consequence of the formation by oxidative coupling of dimers and compounds of higher molecular-weight (46).

After 1 h incubation, 59% of initial EGCG and 76% of monoglucoside ***2*** had disappeared, in contrast with only 11% of the monoglucoside at 3′-OH. In this context, Noguchi et al. reported that the 5-*O*-α-D-glucopyranoside of EGCG was about 1.5-fold more stable than the parent compound at pH 7.0 and 80°C (36). Kitao et al. reported that the α-monoglucoside at C-4′ of B-ring was also substantially more stable than EGCG (37). Therefore, the glycosylation of EGCG in position 3′ of B-ring increases significantly the resistance of EGCG to pH and thermal degradation.

### Toxicity of EGCG Glucosides

**Figure 4:**
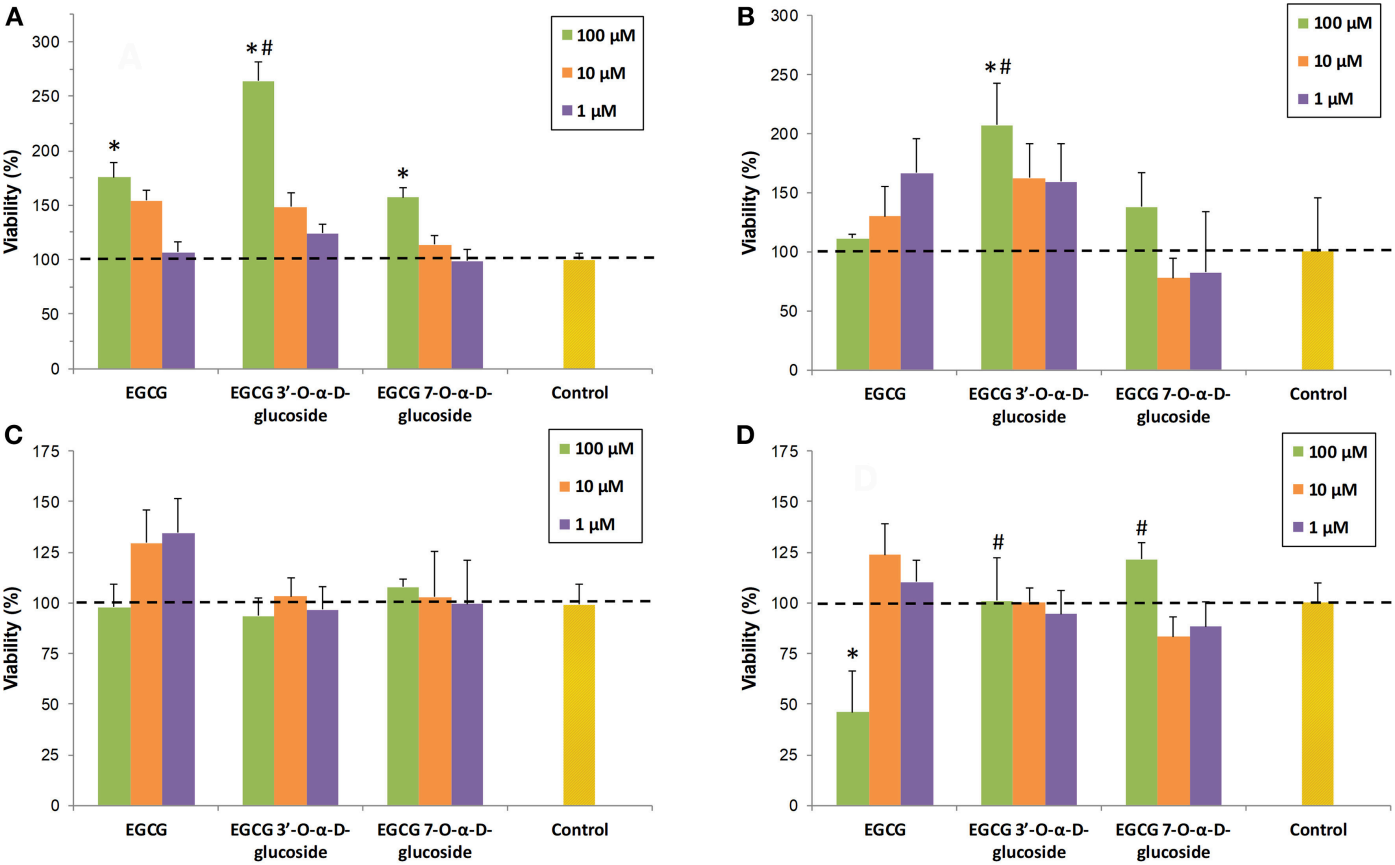
Cell viability assays in presence of EGCG and its α-glucosides ***1*** and ***2*** on: **(A)** SH-SY5Y neuronal cells; **(B)** RAW 264.7 macrophages; **(C)** MRC5 fibroblasts; **(D)** HT-29 colon cancer cells. The values are referred to the control (cells containing 1% DMSO). The data is expressed as mean ± SD (*n* = 8, *p < 0.05 vs. control group; #*p* < 0.05 vs. EGCG (100 μM) group).

The toxicity of EGCG and the isolated monoglucosides ***1*** and ***2*** was tested in four cell lines (human SH-S5Y5 neurons, RAW 264.7 macrophages, MRC5 fibroblasts and HT-29 colon cancer cells). The viability of cells in the presence of the compounds was determined at three concentrations (1, 10, and 100 μM). The final DMSO percentage in each cell was adjusted to 1% (v/v). The values were referred to the control (cells containing 1% DMSO). As shown in Figure 4, EGCG and its glucosides were not significantly toxic for any of the examined cell lines, except for the parent compound EGCG at 100 μM concentration in HT-29 colon cancer cells (Figure 4D).

The cytotoxic effect on HT-29 cancer cells correlates well with previous reports on the specific pro-oxidant action of catechins toward cancer cells (47), which seems to be modulated by sirtuin 3 (SIRT3) (48). Thus, green tea catechins (including EGCG) may exert pro-oxidant activity in cancer cells leading to cell death but antioxidant effects in normal cells (49).

We have observed that the presence of a glucose unit in a natural phenolic compound such as resveratrol, like in piceid (3-β-glucoside of resveratrol), also decreases the intrinsic toxicity of the parent molecule in human embryonic kidney cells (HEK-293) (21). However, this is not a general trend since piceid is more toxic than resveratrol for HT-29 and breast adenocarcinoma MCF-7 cancer cells. The differences in cellular uptake of the compounds could be related to the observed toxicity, especially if the glucose transporters are playing a role in the entrance of the glucoside derivatives.

### ROS and REDOX Activity of EGCG Glucosides

**Figure 5:**
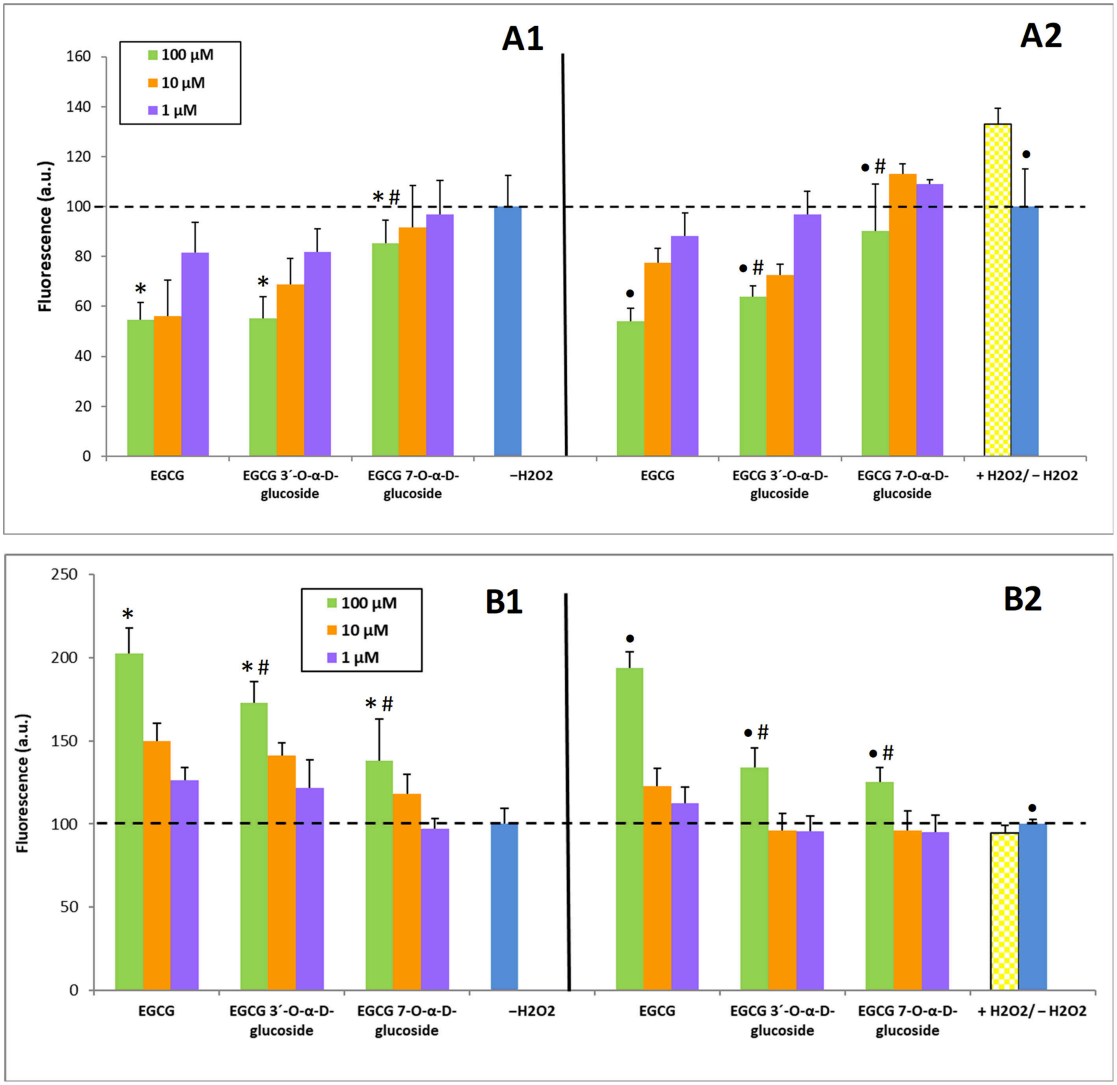
Capacity of EGCG and its α-glucosides ***1*** and ***2*** on SH-SY5Y neuronal cells to: **(A)** Alleviate intracellular ROS levels; **(B)** Enhance intracellular REDOX activity. **(A1, B1)** Incubation (6 h) with the compounds without H_2_O_2_ treatment; **(A2, B2)** Pre-incubation (2 h) with the compounds followed by incubation (2 h) with 100 μM H_2_O_2_. The values are normalized to the experiments in absence of H_2_O_2_ (–H_2_O_2_). The data is expressed as mean ± SD (*n* = 8, *p < 0.05 vs. –H_2_O_2_ group; ^•^*p* < 0.05 vs. +H_2_O_2_ group; #p < 0.05 vs. EGCG (100 μM) group).

Once established the safety of EGCG and EGCG glucosides toward SH-SY5Y neuroblastoma cultures, their potential to alleviate intracellular ROS levels or to boost intracellular REDOX activity was determined (50). The former assays were carried out in the presence of hydrogen peroxide as intracellular ROS trigger. Basal ROS levels (Figure 5A1) were measured from the fluorescence intensity of DCF as it is explained in the Experimental section. As a rule, all compounds produced a dose-response decrease in ROS levels, but this effect was significantly greater for EGCG and EGCG 3′-*O*-α-D-glucoside, compared to EGCG 7-*O*-α-D-glucoside (Figure 5A2). Remarkably, the treatment with 100 μM EGCG and its 3′-α-D-glucoside lowered ROS levels to nearly 50% of the non-stimulated cells value.

Regarding REDOX activity, H_2_O_2_ treatment led to a small decrease of REDOX compared to control cells, which was attenuated by a 100 μM pretreatment with all the compounds screened (Figure 5B2). Bigger differences were observed in REDOX activity between control cells and pretreatment with each compound alone for 6 h, where all the derivatives at 100 μM were able to increase the basal REDOX activity regardless the treatment concentration (Figure 5B1).

### Neuroprotective Activity of EGCG Glucosides

EGCG has arisen a lot of interest as a potential therapeutic agent in the prevention of neurodegenerative diseases (51–53). This ability is related with its antioxidant, radical scavenging, anti-apoptotic and anti-inflammatory properties (54). Several studies confirmed the potential of EGCG to promote healthy aging, suppress cognitive dysfunction, increase learning ability and minimize oxidative damage in the brain (55, 56).

**Figure 6:**
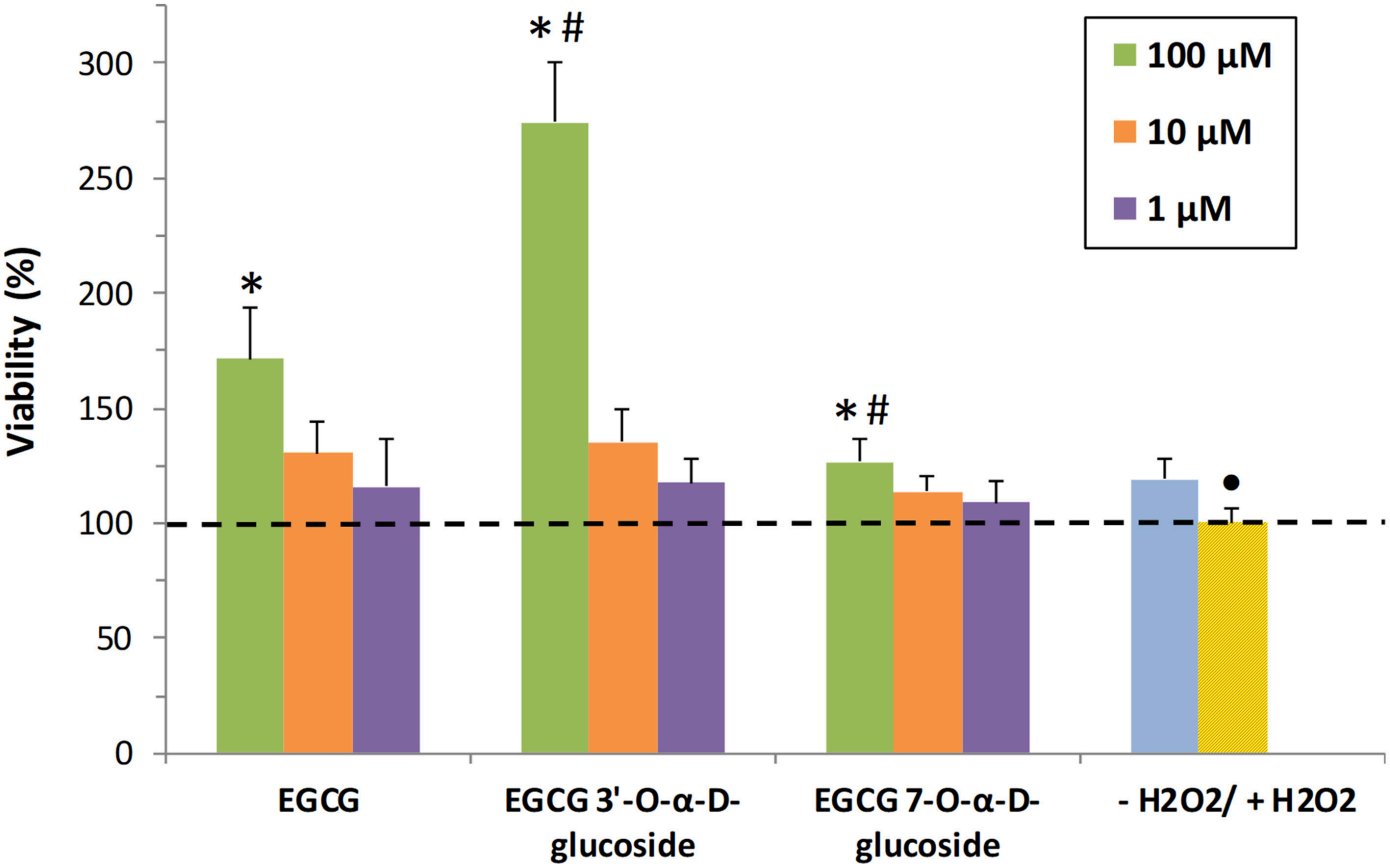
*In vitro* analysis of neuroprotective activity of EGCG and its α-glucosides ***1*** and ***2*** on SH-SY5Y neuronal cells. The values are referred to the viability of cells in presence of H_2_O_2_ (+H_2_O_2_). The data is expressed as mean ± SD (*n* = 8, *p < 0.05 vs. +H_2_O_2_ group; •*p* < 0.05 vs. -H_2_O_2_ group; #*p* < 0.05 vs. EGCG (100 μM) group).

In the present work, the neuroprotective activity of EGCG and the synthesized monoglucosides ***1*** and ***2*** toward human SH-S5Y5 neurons was tested *in vitro*. Previously we demonstrated that EGCG and its glucosides were not toxic for the cells (Figure 4A). Then, the neuroprotective activity in the presence of H_2_O_2_ was tested at the same compound concentrations (1, 10, and 100 μM) (Figure 6). Values above 100% indicated neuroprotection. EGCG and its glucoside ***1*** showed a dose-dependent behavior increasing cells viability after exposure to hydrogen peroxide. In particular, the viability increased 2.75-fold, referred to the cells treated with H_2_O_2_, in the presence of 100 μM of the 3′-glucoside, whilst EGCG increased 1.7-fold the viability of cells. This increased neuroprotection of monoglucoside ***1*** compared to EGCG might be related with their similar antioxidant activity (Figure 2) but the slower degradation of the 3′-glucoside (Figure 3). The enhancement of neuroprotective activity upon glycosylation was more significant than the reported with other related polyphenols such as hydroxytyrosol (13).

Both EGCG and its 3′-α-D-glucoside exhibited better properties at 100 μM than the α-glucoside at C-7 of the A-ring (compound ***2***). This result could be related with the lower antioxidant activity of the C-7 monoglucoside compared with EGCG and its derivative at C-3′ (Figure 2). In this context, Xiao recently reported that several polyphenols with catechol or pyrogallol structure were unstable in cell culture medium such as DMEM in the absence of cells (57). For that reason, the different stability of EGCG and its glucosides (Figure 3), and in particular the stabilization effect upon glycosylation at C-3′, could play a critical role in the bioactivity results presented in this work.

## Conclusion

Two α-glucosides of EGCG were enzymatically synthesized and their properties assayed. The major product ***1*** contained a glucosyl moiety at C-3′ in the B-ring and the minor compound ***2*** was glucosylated at C-7 of A-ring. The compound ***1*** exhibited more interesting properties than ***2***. Thus, it displayed higher pH and thermal stability than EGCG, and a similar radical scavenging activity. It is remarkable that the viability of H_2_O_2_-treated human neurons increased 2.75-fold in the presence of monoglucoside ***1***, whilst EGCG only produced a 1.7-fold enhancement. In conclusion, the α-glucoside of EGCG at C-3′ could be useful for nutraceutical, cosmetic and biomedical applications. However, to determine its full potential, further studies regarding the bioavailability and *in vivo* activity are necessary.

## Author Contributions

FP, JM, and AB conceived and designed the experiments. JG-A and PP performed most of the experiments. FP and JM wrote the paper, which was improved by the rest of authors.

### Conflict of Interest Statement

The authors declare that the research was conducted in the absence of any commercial or financial relationships that could be construed as a potential conflict of interest.
